# Presymptomatic breast cancer in Egypt: role of *BRCA1 *and *BRCA2 *tumor suppressor genes mutations detection

**DOI:** 10.1186/1756-9966-29-82

**Published:** 2010-06-25

**Authors:** Safinaz S Ibrahim, Elsayed E Hafez, Mervat M Hashishe

**Affiliations:** 1 Biochemistry Department, Faculty of Pharmacy, Cairo University, Cairo, Egypt; 2 Mubarak City for Scientific Research and Technology Applications, Alexandria, Egypt; 3 Department of Human Genetics, Medical Research Institute, University of Alexandria, Alexandria, Egypt

## Abstract

**Background:**

Breast cancer is one of the most common diseases affecting women. Inherited susceptibility genes, *BRCA1 *and *BRCA2*, are considered in breast, ovarian and other common cancers etiology. *BRCA1 *and *BRCA2 *genes have been identified that confer a high degree of breast cancer risk.

**Objective:**

Our study was performed to identify germline mutations in some exons of *BRCA1 *and *BRCA2 *genes for the early detection of presymptomatic breast cancer in females.

**Methods:**

This study was applied on Egyptian healthy females who first degree relatives to those, with or without a family history, infected with breast cancer. Sixty breast cancer patients, derived from 60 families, were selected for molecular genetic testing of *BRCA1 *and *BRCA2 *genes. The study also included 120 healthy first degree female relatives of the patients, either sisters and/or daughters, for early detection of presymptomatic breast cancer mutation carriers. Genomic DNA was extracted from peripheral blood lymphocytes of all the studied subjects. Universal primers were used to amplify four regions of the *BRCA1 *gene (exons 2,8,13 and 22) and one region (exon 9) of *BRCA2 *gene using specific PCR. The polymerase chain reaction was carried out. Single strand conformation polymorphism assay and heteroduplex analysis were used to screen for mutations in the studied exons. In addition, DNA sequencing of the normal and mutated exons were performed.

**Results:**

Mutations in both *BRCA1 *and *BRCA2 *genes were detected in 86.7% of the families. Current study indicates that 60% of these families were attributable to *BRCA1 *mutations, while 26.7% of them were attributable to *BRCA2 *mutations. Results showed that four mutations were detected in the *BRCA1 *gene, while one mutation was detected in the *BRCA2 *gene. Asymptomatic relatives, 80(67%) out of total 120, were mutation carriers.

**Conclusions:**

*BRCA1 *and *BRCA2 *genes mutations are responsible for a significant proportion of breast cancer. *BRCA *mutations were found in individuals with and without family history.

## Background

Breast cancer is one of the most prevalent diseases affecting women [1]. In Egypt, breast cancer represents 18.9% of total cancer cases among the Egypt National Cancer Institute during the year 2001 [2]. Breast cancer is the most common cause of cancer related deaths among women worldwide [3]. The etiology of breast cancer involves environmental factors, inherited genetic susceptibility, genetic changes during progression and interaction among these factors, with the relative importance of each ranging from strongly genetic or strongly environmental [4]. In the process associated with the development of breast cancer, it is known that malignant transformation involves genetic and epigenetic changes that result in uncontrolled cellular proliferation and/or abnormal programmed cell death or apoptosis. These cellular abnormalities, i.e. cancer cells; arise through accumulation of mutations that are frequently associated with molecular abnormalities in certain types of genes, such as proto-oncogenes and tumor-suppressor genes, as a result of genetic predisposition and/or exposure to physical, chemical, biological or environmental factors [2]. These mutations are either inherited (germline) or acquired (somatic). Somatic mutation may determine the phenotype of a particular breast cancer and may be of clinical value in determining prognosis. However, only germline mutations can predetermine an individual's risk of developing breast cancer.

Two classes of inherited susceptibility genes are considered in the etiology of breast and other common cancers. First: Genes have been identified that confer a high degree of breast cancer risk. These include *BRCA1, BRCA2 *and *TP*^*53*^. Because of the great effect these genes have on cancer risk, one hallmark of these genes is the creation of a Mendelian autosomal dominant pattern of cancer. These genes also tend to predispose to earlier onset, multifocal breast tumors. Second: Variant genotypes at other loci (polygene) may confer a relatively smaller degree of cancer risk, but they carried by a larger proportion of the general population. In the general population, breast cancer usually occurs in the absence of a strong family history, appears unilaterally, and has a relatively late (often postmenopausal) age at diagnosis [5].

The discovery of breast cancer genes, *BRCAl *and *BRCA2*, has led to an explosive growth in cancer screening for population at risk. Every one carries these genes as part of the normal genetic makeup. Patients who are at risk for breast cancer carry mutations of these genes. Early in the last decades, in 1990, genetic studies provided initial evidence that the risk of breast cancer in some families is linked to position q_2_i of chromosome 17 which was characterized by autosomal dominant inheritance. In fact, loss of heterozygosity at 17q was found in most familial breast and ovarian tumors, suggesting the involvement of tumor suppressor gene(s) [6,7].

In 1994, the breast cancer susceptibility gene *BRCAl*, the most important tumor suppressor gene, was identified by positional cloning. This gene is expressed in numerous tissues, including breast and ovary. *BRCAl *gene is a large gene spread over approximately 100 kb of genomic DNA. It is composed of 24 exons, 1 and 4 are non-coding and are not analyzed, and code for a protein of 1863 amino acids producing a nuclear protein of about 220 kd. It contains a protein motif, a Ring Finger domain near the amino acid terminus and a conserved acidic carboxyl terminus that functions in transcriptional co-activation [6,8]. There is evidence that *BRCA1 *protein being directly involved in the DNA repair process. The *BRCA1 *gene product interacts with the RAD51 protein, a key component in homologous recombination and double strand break repair [9].

In 1995, the *BRCA2 *gene was identified at chromosome 13qi_2_-i3. *BRCA2 *gene is even larger than *BRCA1*, consists of 27 exons, 1 is non-coding and is not analyzed, and codes for a protein of 3418 amino acids, making a 380 kd nuclear protein. *BRCA2 *gene has no obvious homology to any known gene and the protein contains no well-defined functional domain [10]. The *BRCA2 *protein also interacts with RAD51. Perhaps through this mutual association with RAD51, *BRCA1 *and *BRCA2 *associate with each other at sites of DNA synthesis after the induction of DNA damage. Nonetheless, *BRCA1 *and *BRCA2 *proteins appear to share a number of functional similarities that may suggest why mutations in these genes lead to specific hereditary predisposition to breast and ovarian cancer [11]. *BRCA *genes contribute to DNA repair and transcriptional regulation in response to DNA damage and cell cycle control. Recent studies suggest that *BRCA *proteins are required for protecting the genome from damage [12].

Mutations in *BRCA *genes have been established to predispose women to breast and ovarian cancer, the end point of *BRCA *protein dysfunction. Mutations in both genes are spread throughout the entire gene. More than 600 different mutations have been identified in *BRCAl *gene and 450 mutations in *BRCA*. The majorities of mutations, known to be disease-causing, results in a truncated protein due to frame shift, nonsense, or splice site alternations. Nonsense mutations occur when the nucleotide substitution produces a stop codon (TGA, TAA, or TAG) and translation of the protein is terminated at this point. Frame shift mutations occur when one or more nucleotides are either inserted or deleted, resulting in missing or non-functional protein. Splice site mutations cause abnormal inclusion or exclusion of DNA in the coding sequence, resulting in an abnormal protein. Other kind of mutations results from a single nucleotide substitution is missense mutations in which the substitution changes a single amino acid but does not affect the remainder of the protein translation [13,14]. Studies of *BRCAl *mutation occurrence suggested that nearly half of the families at high risk for breast cancer carried *BRCAl *mutation [15]. However, other analysis suggest that the actual incidence of *BRCAl *in high risk families (>3 cases of breast and/or ovarian cancer) might be as low as 12.8% to 16% [4]. Substantial variation in the prevalence of *BRCA1 *mutations in high risk families in various countries has been observed which are more common than *BRCA2 *mutations [16,17].

The main objectives of the present work were to identify germline mutations in *BRCA1 *(exons 2, 8, 13, 22) and *BRCA2 *(exon 9) genes for the early detection of presymptomatic mutation carriers in Egyptian healthy females who were first degree relatives of affected women from families with and without family history of breast cancer.

## Subjects and Methods

### Patients and families

Sixty breast cancer patients **(**index patients), derived from 60 families, considered being at high risk, due to medicinal examination and they were grid 3 patients, were selected for molecular genetic testing of *BRCA1 *and *BRCA2 *genes. They were referred to the Clinical Oncology Unit in Medical Research Institute, Alexandria University, for chemotherapy as part of their curative treatment after mastectomy. Selected index patients were preferred to be at early onset age at diagnosis, possessing a positive family history and bilateral breast cancer. The study also included one hundred and twenty healthy first degree female relatives of index patients either sisters and/or daughters for early detection of mutation carriers. The decision to undergo genetic testing was taken after the participants were informed about benefits and importance of genetic testing. All studied subjects gave written informed consent. Index patients were asked for detailed information on family history of breast or any other cancer type in their families. Our study protocol was approved by the Medical Research Institute, University of Alexandria, Alexandria, Egypt.

### DNA isolation and PCR amplification for the different exons

Blood samples (3 ml each) were collected from the patients (60 women) and the healthy asymptomatic first degree female relatives (120 relatives) in EDTA tubes. Genomic DNA was extracted from peripheral blood lymphocytes using a Promega DNA purification kit (Promega, Madison, USA), following the manufacturer's instructions. Universal primers (Table 1) were used to amplify four regions of the *BRCA1 *gene (exons 2, 8, 13 and 22) and one region of *BRCA2 *gene (exon 9). The polymerase chain reaction (PCR) was carried out using 50 ng of DNA, 10 × PCR buffer with 1.5 mM MgCl_2_, 2 ul of mixture of 4 mM dNTPs, 20 pmol of each primer and 1U of Tag DNA polymerase at final volume of 25 ul. The PCR conditions were 96°C for 5 minutes, then 35 cycles each consists of 30 sec at 94°C, one min at the annealing temperature of the primer used (mostly around 56-59°C) and one min at 72°C, followed by one cycle at 72°C for 10 minutes.

**Table 1:** Primers' sequences employed in the specific-PCR

Primers	Sequence (5'- 3')
**BRCA1** **Exon 2**	Sense: GAAGTTGTCATTTTATAAACCTTT Antisense: GTCTTTTCTTCCCTAGTATGT

**BRCA1** **Exon 8**	Sense: TGTTAGCTGACTGATGATGGT Antisense: ATCCAGCAATTATTATTAAATAC

**BRCA1** **Exon 13**	Sense: AATGGAAAGCTTCTCAAAGTA Antisense: ATGTTGGAGCTAGGTCCTTAC

**BRCA1** **Exon 22**	Sense: ATG TTG GAG CTA GGT CCT TAC Antisense: GAG AAG ACT TCT GAG GCT ACG

**BRCA2** **Exon 9**	Sense: CAT CAC ACT ACT CAG GAT GAC A Antisense: GCA TGG TGG TGC ATG CTT GTA

### **Mutation detection using the Single strand conformation polymorphism assay **(**SSCP)**

SSCP analysis were used to screen for mutations in the exons 2, 8, 13, 22 of *BRCA1 *gene and exon 9 of *BRCA2 *gene in all studied subjects[18,19]. Every PCR product was mixed 1:1 with loading buffer (95% formamide, 0.05% bromophenol blue and 0.05% xylene cyanol), and denature at 98°C for 10 min and suddenly place in ice. Electrophoresis of the denatured PCR products were carried out in 8% polyacrylamide gel containing 5% glycerol and the run was performed at 30 mA constant current for 6 hours. After that, the gel was stained by Ethidium Bromide for minutes, washed by water and visualized using the gel documentation system.

### Mutation detection using heteroduplex analysis

Heteroduplex assay was carried out, as a confirmatory analysis for detecting mutations, in case of families which had no detected mutation in either of the studied exons of both genes by SSCP assay. PCR for the patients and normal samples were carried out using the specific primer of any one of the studied exons. Equal volumes of PCR products (form the patient and normal ones) were equally mixed with loading buffer, the mixture was heated to denature at 95°C/10 min and cooled to room temperature over a period of 2 hours to induce heteroduplex formation. Electrophoresis was done to the mixture through 12% polyacrylamide gel for 6 hours at a constant 60 V. The gel was stained with Ethidium Bromide for 30 seconds and visualized on the gel documentation system. Any heteroduplex migrate more slowly through the gel as compared to its homoduplex counter parts. Sequence change could be detected by an extra band above the main homoduplex band.

### DNA sequencing of normal and mutated exons

PCR samples showing variant bands as well as that of normal subjects were analyzed by direct DNA sequencing technique.

## Statistical analysis

The data, either clinical or genetic findings, were statistically evaluated, interpreted and analyzed using the SPSS software version 16.

## Results

### Detected mutations

**Figure 1:**
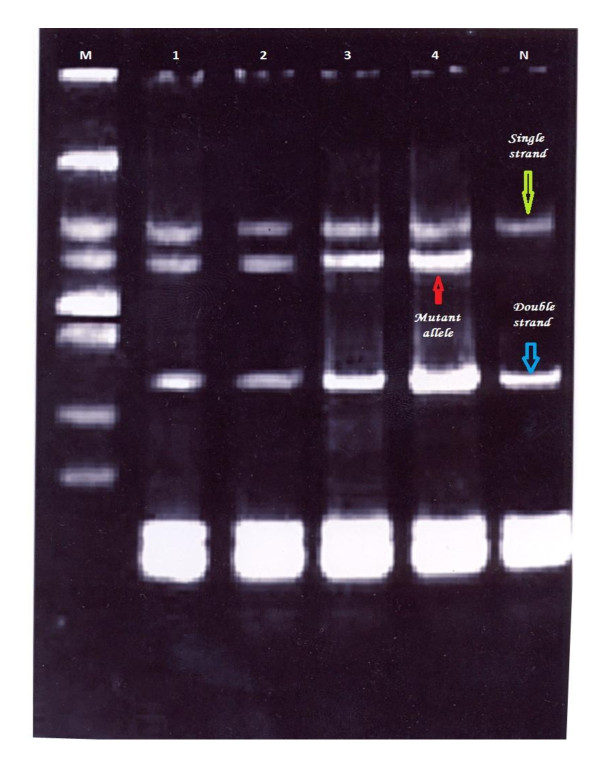
**Single strand conformation polymorphism (SSCP) assay for exon 2 (BRCA 1) germline mutations**. Lane N, normal female control. Lanes 1, 2, 3 and 4 show abnormal pattern of SSCP for patient, her sister and her daughters. Lane M, 50 bp DNA ladder.

**Figure 2:**
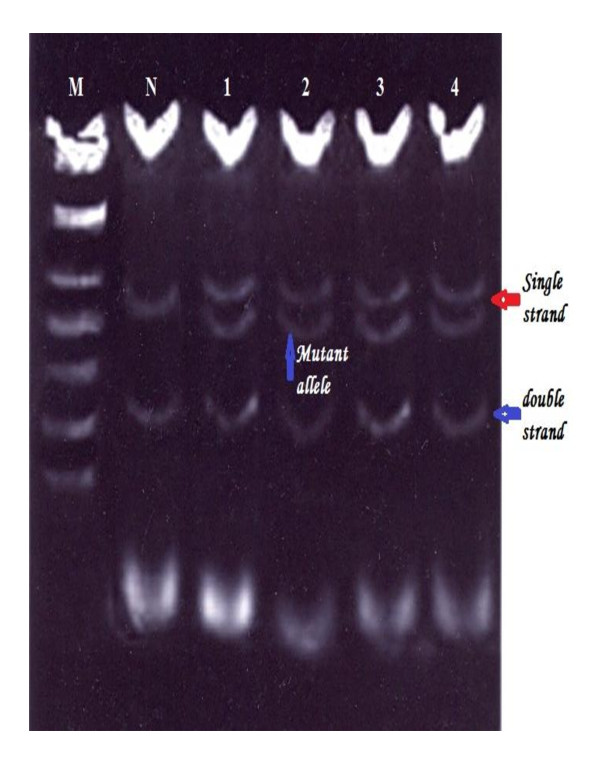
**Single strand conformation polymorphism (SSCP) assay for exon 22 (BRCA 1) germline mutations**. Lane N, normal female control. Lanes 1, 2, 3 and 4 show abnormal pattern of SSCP for patient, her sister and her daughters. Lane M, 50 bp DNA ladder.

**Figure 3:**
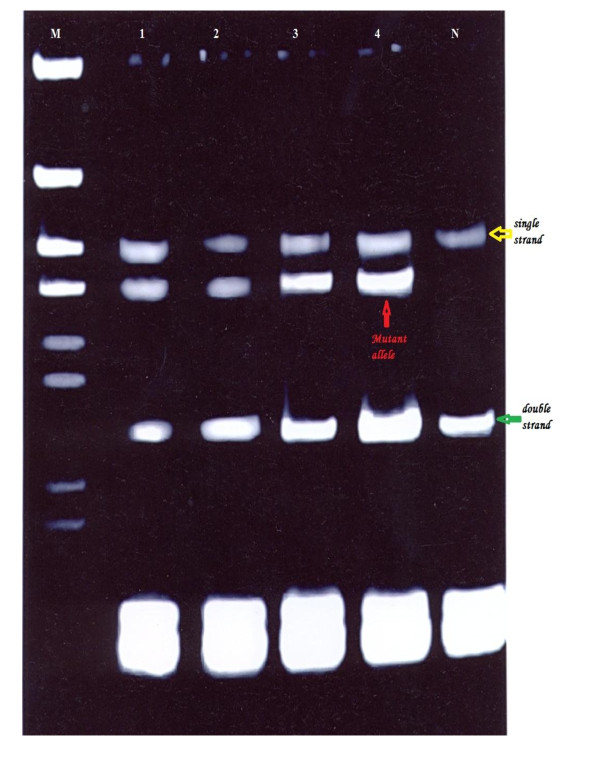
**Single-strand conformation polymorphism assay for exon 13 (BRCA 1) germline mutations**. Lane N, normal female control. Lanes 1, 2, 3 and 4 show abnormal pattern of SSCP for patient, her sister and her daughters. Lane M, 50 bp DNA ladder.

**Figure 4:**
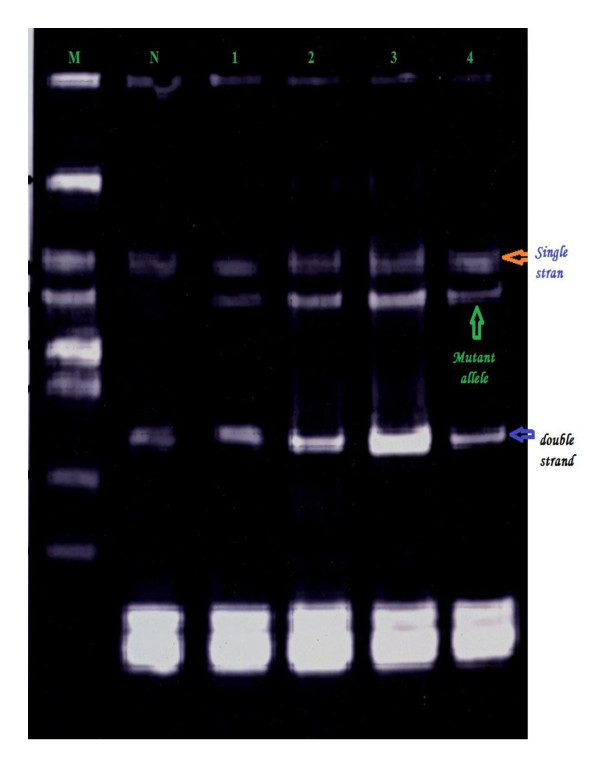
**Single-strand conformation polymorphism assay for exon 8 (BRCA 1) germline mutations**. Lane N, normal female control. Lanes 1, 2, 3 and 4 show abnormal pattern of SSCP for patient, her sister and her daughters. Lane M, 50 bp DNA ladder.

**Figure 5:**
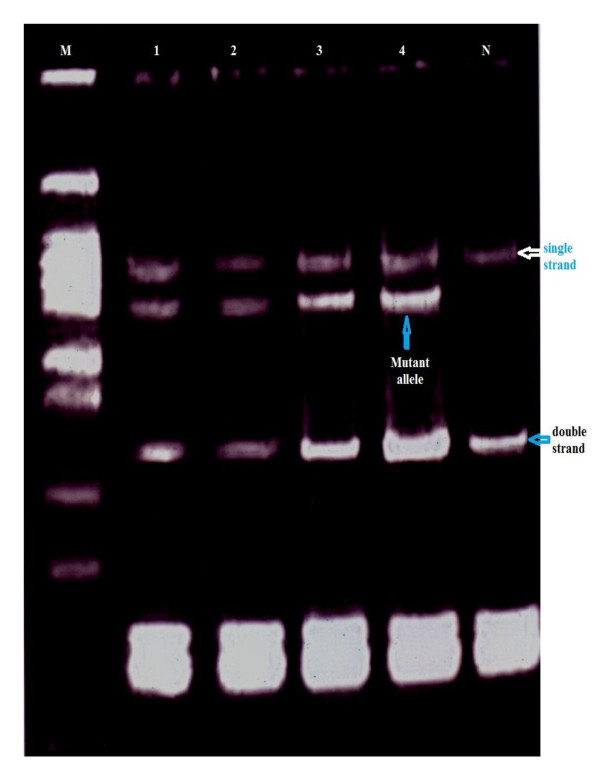
**Single-strand conformation polymorphism assay for exon 9 (BRCA 2) germline mutations**. Lane N, normal female control. Lanes 1, 2, 3 and 4 show abnormal pattern of SSCP for patient, her sister and her daughters. Lane M, 50 bp DNA ladder.

**Figure 6:**
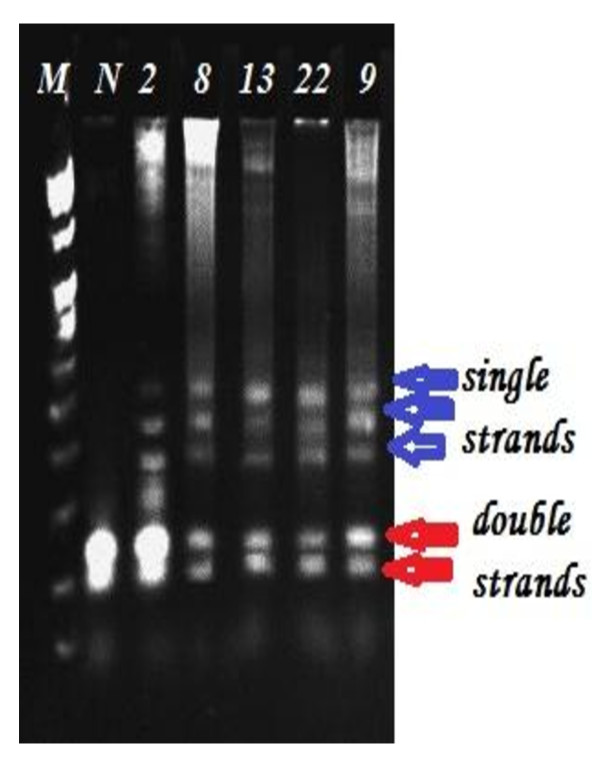
**Shows Heteroduplex analysis for germline mutations**. Lane M marker, Lane 1 shows normal pattern, Lanes 2-5 BRCA1 abnormal patterns, Lane 2 exon 2 patients, Lane 3 exon 8 patient, Lane 4 exon 13 patient, Lane 5 exon 22 patient, Lane 6 BRCA2 exon 9 abnormal pattern

**Figure 7:**
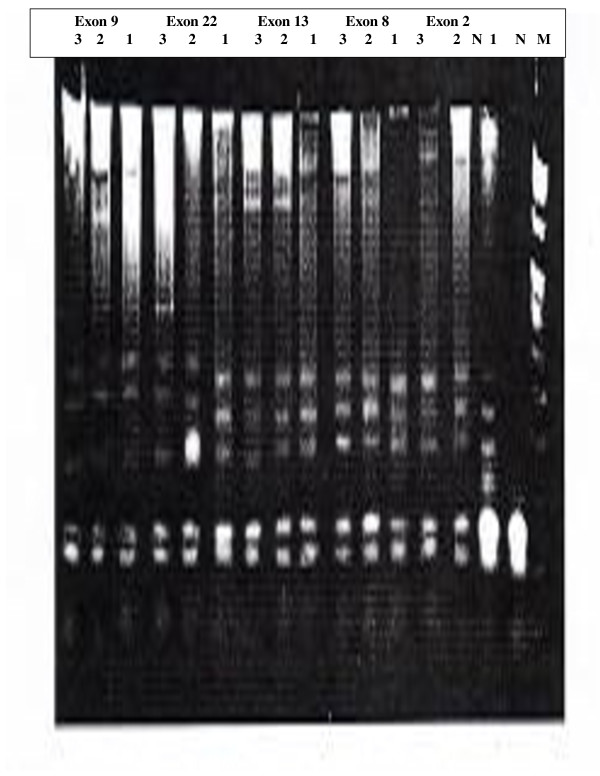
**Shows Heteroduplex analysis for germline mutations in both BRCA genes (BRCA1 exons 2, 8, 13, 22 and BRCA2 exon 9; double heterozygote), inherited in a family**. Lane M marker, Lane N normal control, Lane 1 for patient, Lane 2 and 3 for her daughters, in every exon.

Mutations were detected in 86.7% of the families (52 from total 60 families), in either *BRCA1 *or *BRCA2*. Of them 60% families were attributable to *BRCA1 *mutation and 26.7% families were attributable to *BRCA2 *mutations. They were identified by using the combination of SSCP (Figures 1, 2, 3, 4 and 5) and heteroduplex analysis (Figures 6, 7). Four mutations were detected within the *BRCA1 *gene, and one mutation was detected in the *BRCA2 *gene. Eighty, from the total 120, asymptomatic relatives were mutation carriers.

### DNA sequencing of normal and mutated exons

Results showed that there is difference in nucleotide sequence between the normal and mutated exons. The detected *BRCA1 *mutations comprised four distinct alterations distributed across the coding sequence of the gene. Two were frame shift mutations localized to exon 2 (185 del AG) and exon 22 (5454 del C) (Table 2), one nonsense mutation localized to exon 13 (4446 C--T) and one missense mutation in exon 8 (738 C- -A). The *BRCA 2 *mutation was frame shift mutation localized to the studied exon 9 (999 del 5) (Table 3).

**Table 2:** Sequencing data of exon 22 of BRCA1 gene which amplified from healthy woman (control) and patient with breast cancer, the alignment was carried out using Clustal W 1.9 program.

Subject	Nucleotide sequence	Number
**Control**	**TGAAACCTGCCCTAATAATTCAGTCATCTCTCAGGATCTTGATTATAAAGAAGCAAAATG**	60
**Patient**	TGAAACCTACCTTTATAACTTAGTCCAATCTCTAGATTTTGATTTTAAAGAAACAAATAG ******** ** * **** * **** **** *** ****** ******* **** *	60

**Control**	**TAATAAGGAAAAACTACAGTTATTTATTACCCCAGAAGCTGATTCTCTGTCATGCCTGCA**	120
**Patient**	TAATAAGGAAAAACTACAGTTATTTATTACCCCAGAAGCTGATTCTCTGTCATGCCTGCA ************************************************************	120

**Control**	**GGAAGGACAGTGTGAAAATGATCCAAAAAGCAAAAAAGTTTCAGATATAAAAGAAGAGGT**	180
**Patient**	**GGAAGGACAGTGTGAAAATGATCCAAAAAGCAAAAAAGTTTCAGATATAAAAGAAGAGGT** ************************************************************	180

**Table 3:** Sequencing data of exon 9 of BRCA2 gene which amplified from healthy woman (control) and patient with breast cancer, the alignment was carried out using Clustal W 1.9 program.

Subject	Nucleotide sequence	Number
**Control** **Patient**	**ATCACACTTCTCAGGATGACCCATCAGGTATTCTGATTCACCAAAGCGACTCATGGATAA** **||||||||||||||||||||||||||||||||||||||||||||||||||||||||||||** **ATCACACTTCTCAGGATGACCCATCAGGTATTCTGATTCACCAAAGCGACTCATGGATAA**	1-60 1-60

**Control** **Patient**	**GGGGGGACTACTACTATATGTGCATTGAGAGTTTTTATACTAGTGATTTTAAACTATAAT** **||||||||||||||||||||||||||||||||||||||||||||||||||||||||||||** **GGGGGGACTACTACTATATGTGCATTGAGAGTTTTTATACTAGTGATTTTAAACTATAAT**	61-120 61-120

**Control** **Patient**	**TTTTGCAGAATGTGAAAAGCTATTTTTCCAATCATGATGAAAGTCTGAAGAAAAATGATA** **||||||||||||||||||||||||||||||||||||||||||||||||||||||||||** **TTTTGCAGAATGTGAAAAGCTATTTTTCCAATCATGATGAAAGTCTGAAGAAAAATGATA**	121-180 121-180

**Control** **Patient**	**GATTTATCGCTTCTGTGACAGACAGTGAAAACACAAATCAAAGAGAAGCTGCAAGTCATG** **||||||||||||||||||||||||||||||||||||| ||||||||||||||||||** **GATTTATCGCTTCTGTGACAGACAGTGAAAACACAAA-----GAGAAGCTGCAAGTCATG**	181-240 181-235

**Control** **Patient**	**GTAAGTCCTCTGTTTAGTTGAACTACAGGTTTTTTTGTTGTTGTTGTTTTGATTTTT** **|||||||||||||||||||||||||||||||||||||||||||||||||||||||||** **GTAAGTCCTCTGTTTAGTTGAACTACAGGTTTTTTTGTTGTTGTTGTTTTGATTTTT**	241-297 236-292

### Mean age at diagnosis

The mean age at diagnosis of breast cancer in *BRCA1 *mutation carriers was 42.4 years while in *BRCA2 *mutation carriers was 34.3 years. The mean age at diagnosis of breast cancer in *BRCA *mutation carriers and non carriers were 39.8 years and 47.1 years respectively.

### Family history

Positive family history was found in 39 (65%) families (included 39 patients their ages at diagnosis ranged from 23 to 45 years). Pathologic mutations were detected in 35 families, in 4 families of them, the affected index cases and their 1^st ^degree relatives were mutation carriers for both *BRCA1 *and *BRCA2 *gene. Negative family history patients included a group of 21 women diagnosed with breast cancer belonging to 21 families (35%). Of them 15 women included in 15 (25%) families their ages at diagnosis ranged from 18 to 40 years. Germline mutations in predisposing *BRCA1 *gene were detected in these women and their daughters. In addition, 2 (3.3%) families in which the index patients had bilateral breast cancer diagnosed at ages 44 and 49 years with negative family history found to have mutation in *BRCA1 *gene.

### Pedigree characteristics

Most index cases, which have a family history of breast cancer, lack the pedigree characteristics of autosomal dominant inheritance of cancer predisposition. Example of pedigree with positive family history shows the proband's sister and their mother are affected and one of her daughters is also affected, the other asymptomatic daughter of the proband is mutation carrier by DNA testing. This mutation carrier female has two daughters on testing one is mutation negative and the other is mutation carrier. Example of pedigree with no family history shows that the patient (proband) aged 32 years at onset of breast cancer have 3 daughters and three normal sisters. One of the asymptomatic daughters on testing found to be mutation carrier for *BRCAl *gene. In addition, the grand daughter of this proband is also mutation carrier.

## Discussion

Efforts are underway to reduce the high incidence and mortality associated with breast cancer, which can be achieved by the early detection of women at high risk. Since genetic predisposition is the strongest risk factor, molecular testing can be considered as the only way for early detection of breast cancer. DNA testing for breast cancer susceptibility became an option after the identification of the *BRCA1 *and *BRCA2 *genes. Germline mutations in either of the two predisposing genes, *BRCA1 *and *BRCA2*, account for a significant proportion of hereditary breast cancer [14]. Women with either *BRCA *mutation have a cumulative lifetime risk of invasive breast cancer of about 55-85% [20]. Generally, it has not been possible for clinician to determine which individual in a high risk families are carriers of *BRCA *mutations. Women, who may not have these mutations, may have undergone unnecessary intervention including prophylactic surgery. So the availability of the *BRCA *analysis has beneficial impact on the care and counseling of women at risk [4]. Analysis of *BRCA1 *and *BRCA2 *genes makes it possible to identify predisposing mutations in affected persons and determine risks for family members. Once a mutation is identified in affected person, asymptomatic family members can be tested for this only mutation, since first degree relatives of patients with breast cancer have increased risk for early onset of this cancer [21,22].

All the studied Egyptian patients had invasive breast carcinoma. From them, 39 patients had both positive family history and early age at onset and a sample composed of 15 (25%) patients had no family history but had early onset of the disease. The occurrence of BRCA mutations in this 25% of the studied patients, with early onset and no family history of breast cancer, suggests that the age at diagnosis in patients with negative family history is an important indicator for the presence of pathologic mutations and lends support to the screening of *BRCA *genes in patients with early onset of the disease. This finding is nearly similar to a study in a group of Czeck women (12.9%) with early onset non-familial breast cancer [14]. In contrast to this, only 2% of non-familial patients had pathologic germline mutations in *BRCA1 *and 2 genes in a group of English patients who were diagnosed with breast cancer at the age of 30 years or younger [23]. So the absence of correlation between family history and the genetic risk attributable to *BRCA *genes could reflect variation in family structure and influence of additional modifier genes [24].

Although *BRCA1 *and *BRCA2 *genes exhibit profound allelic heterogeneity, a large number of repeated mutations have reported, some of them represent founder mutations. The knowledge of founder mutations can shorten the search for an inherited disease-associated mutation. So, in geographic areas where breast cancer population genetics has not yet been widely studied, founder mutations can provide a starting place for understanding of the public health impact of inherited predisposing genes [25]. Ethnicity plays a role in hereditary breast cancer through its association with particular founder mutations. Founder mutations in populations with different national groups have been described in Ashkenazi Jews, Icelanders, French and other populations [26-29]. With knowledge of these mutations, it may be better to screen for a small number of founder *BRCA *mutations in all early onset cancer cases, rather than to attempt comprehensive mutation screening for the minority of cases with a strong family history [30].

The purpose of doing *BRCA *testing in the current study was to find and examine the biodiversity of a mutations in families of patients with breast cancer for the aim of early detection of presymptomatic relatives who are carriers for mutation. It is difficult to identify all mutations in these large genes, so we searched for mutations in certain exons of *BRCA 1 *and *2 *genes. These exons contain frequently recurring mutations described worldwide, but the type of mutations identified can differ considerably from country to country. Only few mutations are dispersed world wide [31]. In the present study we searched for three founder mutations in exons 2, 22, of *BRCA1 *gene, and 9, of *BRCA2 *gene. In addition, two middle regions (exons 8 and 13) of *BRCA1 *gene were investigated for the presence of mutation.

The majorities of mutations, known to be disease-causing, consist of small frame shift deletions, small insertions and nonsense or splice site mutations, which all result in a truncated protein. Because of the lack of known structure-function relationships, only truncating mutations are usable for medical management of carrier individuals [14]. In the current study four truncating mutations and one missense mutation were detected among the majority of the studied patients and in more than half of their asymptomatic first degree female relatives. The truncating mutations were three frame shift mutations and one nonsense mutation. All mutations were repeated in 6 or more families. The recurrent mutations were found in all (100%) families with detected mutations. This finding is similar to the study of Corski *et al*. [32], which found recurrent mutations in 93% of families with detected mutations.

The first studied founder mutation in the current study was the frame shift mutation 185 del AG in exon 2 of *BRCA1 *gene. It was identified in 10% of families (index cases and their asymptomatic relatives). This mutation was detected with high frequency in Ashkenazi Jews [33], in two Spanish families [34], in 3 of 4 families with Ashkenazi Jewish ancestry in France [35] and in non-Ashkenazi groups across the middle east, Turkey, England, Iran, Asia and India [33,36].

The second studied founder mutation in *BRCA1 *gene is a frame shift mutation in exon 22 (5454 del C). It is recently detected in 16.7% Filipino patients and their asymptomatic relatives [28]. The knowledge about this mutation is limited [29]. The third studied founder mutation in *BRCA2 *gene is the frame shift (5-base deletion) mutation in exon 9 (999 del 5). This mutation is recurrent and proposed as an ancient founder mutation. It has been identified as a strong founder in Iceland [37,38]. Also it was identified in Finnish breast cancer families [39], which may reflect ancient genetic relationships between European populations. Other *BRCA2 *founder mutations in other exons have been reported in Filipino patients [28], and in Jewish patients [40]. In the present study, *BRCA2 *mutation is frequently repeated among different families (26.7%) in both patients and their relatives, suggesting a founder effect in our population. The presence of this mutation is not limited to those patients having a positive family history of the disease. Some patients carrying this mutation have negative family history. Failure to identify family history may be attributed to small family size and young relatives. For *BRCA2*, a study [39] has provided evidence that mutation in a ~3.3 kb nucleotide region of exon 11 (denoted the ovarian cancer cluster region, OCCR) is associated with a higher incidence of ovarian cancer relative to breast cancer. So, there is a suggestion that mutation in OCCR is less penetrate for breast cancer at younger ages. In the current study, the *BRCA2 *mutation in exon 9 is outside the OCCR. This explains why all the Egyptian breast cancer patients having this mutation are of young age, less than forty.

In our study, the identified repeated mutation in exon 13 of *BRCA1 *gene is a nonsense mutation (4446 C--T). It was detected in 20% of families. This mutation was found frequently in French-Canadian families and two families in France [35]. These multiple instances of mutation did not represent a founder effect many generations in the past. There was evidence for multiple independent *BRCA1 *mutational events and so multiple origins [41]. The 4446 C--T mutation is one of the most common mutations found in the Breast Cancer Information Core Data base. These mutations are likely to have arisen independently owing to the presence of mutational hot spots in the coding sequence of the gene [42].

The last investigated exon in *BRCA1 *gene for detection of mutation was exon 8. It has been found that 13.3% of index patients and half their asymptomatic relatives have mutation in exon 8(738 C--A). This mutation is a missense mutation predicted to destroy the protein ring-finger. Hamann *et al*. [37] found one missense mutation in exon 8 of *BRCA1 *gene in Germany.

The coexistence of more than founder mutation has been reported in some Ashkenazi Jewish families [40]. In the current study, four families of the 60 Egyptian families were found to have inherited mutation in both *BRCA1 *and *BRCA2 *genes, they are double heterozygote. Previous studies described an Ashkenazi Jewish patient found to have germline mutations in both *BRCA1 *and *BRCA2 *genes [43]. The potential explanation for the occurrence of the two mutations occurring in the same individual is that *BRCA1 *and *BRCA2 *have been implicated in the maintenance of genomic integrity [9,11].

Collectively, it is obvious that *BRCA1 *and/or *BRCA 2 *mutations have been found to account for a greater proportion of breast cancer patients among the studied families. This observation might be due to the relatively young ages of diagnosis of breast cancer and that the hereditary cancers occur disproportionally in young women. The accumulation of *BRCA1 *and *BRCA2 *mutations data from sets of families revealed the prevalence of different mutations and the significance of the putative recurrent founder mutations in Egyptians. The high frequency of any recurrent mutation (frame shift), so far, suggest that there may be a strong *BRCA1 *and *2 *founder effects in Egyptian population. The presence of putative founder mutations, which leading to reduce genetic heterogeneity of *BRCA *genes, facilitates carrier detection and genetic counseling. Therefore, in countries, where founder mutations are present, it is more economical to screen all cases for founder mutations than do perform a complete screen for the two genes on a subset of familial cases. Mutations analysis for a limited set of founder mutations requires much less time, resources and labor than complete screening of genes, resulting in a significant reduction in cost per mutation detected, and a greater number of mutations will be found.

In the present study, eight index cases and their families showed negative results (i.e. no detected mutation in *BRCA1 *or *BRCA2*). This can be explained on the basis that, there may be no inherited predisposition to the disease. In addition, failure to detect a mutation does not exclude the possibility that the individual has predisposing *BRCA1 *or *BRCA2 *mutation as we did not screen the whole gene. These families, in whom no *BRCA *mutations have been identified in the proband, have no risk of passing the mutation to their off spring and can be considered to have breast cancer risk equal to that of the general population, if there is no evidence for a breast cancer gene inherited from the other side of the family (paternal side) [4]. It is appropriate to offer mutation analysis to both parents of an individual with a *BRCA *cancer predisposing mutation. In our study, four affected index cases had mutation in *BRCA1 *gene, their mothers were died from breast cancer before the beginning of the genetic testing, and they might be obligate carriers for *BRCA1 *mutation. For sisters of an index case, the risk depends on the genetic status of the index case's parents. The risk that a sister of an index case will inherit the *BRCA1 *or *BRCA2 *mutation is 50%, if their mother has the mutation. The risk of developing cancer, however, depends upon variables including the peretrance of the mutation, and age of the individual. The *BRCA *genes are highly peretrant and the studied females had a young age at onset of breast cancer. For daughters of an index case identified as having *BRCA1 *or *BRCA2 *mutations, they have a 50% chance of inheriting the mutation.

Counseling was offered to each of the studied family. Women who not likely to have inherited a *BRCA *mutation understand that they remain at risk of developing sporadic breast cancer at a rate roughly equivalent to that of the general population. Women with putative inherited *BRCA *mutations confer an increased risk of developing breast cancer [44]. For counseling of women identified as having a double heterozygote for mutations in *BRCA1 *and *BRCA2*, the risk of transmitting a breast cancer susceptibility gene to any daughters is ¾ [45]. Asymptomatic relatives who test negative for the specific mutation (i.e. do not carry the mutation found in the index cases) are at no increased risk by being related to carriers and have no risk of passing the mutation to their off spring.

## Conclusion

*BRCA1 *and *BRCA2 *genes mutations are responsible for a significant proportion of breast cancer. *BRCA *mutations were found in individuals with and without family history.

## Recommendations

Treatment of advanced breast cancer is often useless and disfiguring, making early detection is of high priority in medical management of the disease. Molecular testing is the only way for early detection of breast cancer. Mutational analysis for a limited set of founder mutations requires much less time, resources, and labor than complete sequencing. Recommendations can be made for public health action on molecular genetic testing. The increased public awareness of the nature and prevalence of breast cancer may result in an increased demand for genetic testing for breast cancer susceptibility. It is valuable to offer genetic testing to newly diagnosed cases with breast cancer for the purpose of clinical management and as a mean to identify presymptomatic carrier relatives for prevention.

## Competing interests

The authors declare that they have no competing interests.

## Authors' contributions

*SSI: *Participated in the design of the study; carried out the molecular genetic studies; drafted the manuscript; revised and approved the final manuscript. *EEH*: Participated in the design of the study; carried out the molecular genetic studies; performed the statistical analysis; read and approved the final manuscript. *MMH*: Participated in the design of the study; selected the patients; collected the samples; read and approved the final manuscript.
